# Lymphatic Transport Efficiency Determines Metastatic Potential of Cutaneous Melanoma

**DOI:** 10.3389/fonc.2020.01607

**Published:** 2020-09-11

**Authors:** Ashley M. Holder, Arturas Ziemys

**Affiliations:** ^1^Department of Surgery, Houston Methodist Hospital, Houston, TX, United States; ^2^Department of Nanomedicine, Houston Methodist Research Institute, Houston, TX, United States; ^3^Department of Surgery, University of Alabama at Birmingham, Birmingham, AL, United States

**Keywords:** melanoma, metastasis, lymphatics, transport, lymph node

## Abstract

**Background:**

In staging patients with clinical stage I-II melanoma, the sentinel lymph node (SLN) is the most important prognostic indicator; however, the false negative rate of SLN biopsy (SLNB) is 15%.

**Methods:**

Nine patients with clinical Stage I-II melanoma underwent SLNB with repeated intraoperative radiotracer measurements to determine lymphatic transport efficiency (LTE), which was correlated with clinicopathologic data.

**Results:**

LTE demonstrated the potential to predict SLN status. LTE in patients with occult nodal metastasis is 40 times faster than those with negative SLNBs. There was no confounding of LTE by clinicopathologic factors.

**Significance:**

LTE may be a novel biomarker for metastasis, with transformative potential for personalized precision diagnostics of early-stage disease and improved patient survival.

## Introduction

Cutaneous melanoma without known metastases is staged and treated by excision of both the primary tumor as well as sentinel lymph node biopsy (SLNB). However, the false negative rate is 15% at major academic centers (1), and the negative predictive value of SLNB is 88% in patients with thick, ulcerated melanomas, despite these patients being at highest risk for metastasis (2). Delays in adjuvant therapy and decreased survival occur in those with false negative SLNB (3). We propose that the accuracy of metastatic prediction and ultimately patient outcomes can be improved through a novel intraoperative technique designed to measure lymphatic transport efficiency (LTE).

The lymphatics are the principal metastatic escape route from primary melanoma tumors; thus, we hypothesized that the lymphatics, which connect the primary tumor with tumor-draining lymph nodes, are likely to influence escape of the tumor. The risk of metastatic disease in melanoma is more strongly associated with lymphatic transport than with Breslow thickness and ulceration (4). Peritumoral lymphatic density is twice as high around primary melanoma in patients who developed metastases and has no relationship to Breslow thickness or ulceration, which are currently used for prognosis and need for SLNB (4). In addition, physical changes in the lymphatic system have been observed before nodal metastases. These alterations include increased lymphangiogenesis (5), lymph node enlargement (6), and lymphatic vessel dilation (7). Lymphatic flow rate also increases through tumor-draining nodes compared to non-tumor draining lymph nodes in mice (5), thereby increasing cancer cell delivery to lymph nodes (8).

Once cancer cells infiltrate, the efficiency of metastasis relies on LTE, determined by diffusion and flow properties of the tumor-draining lymphatic system. Therefore, lymphatic density should determine the throughput, whereas LTE should govern the kinetics and efficiency of metastatic escape from the primary tumor.

## Materials and Methods

### Patient Enrollment and Clinicopathologic Data Collection

Nine patients with clinically non-metastatic cutaneous melanoma who qualified for SLNB were enrolled in an IRB protocol (HMH Pro00021626, PI = Holder) to undergo intraoperative measurement and clinicopathologic data collection[Age, Sex, BMI, Primary tumor location (Head and neck, trunk, and extremity), Breslow thickness and ulceration of primary tumor, number and status of SLN(s), dose of technetium-99m sulfur colloid (TC-99m SC)] during standard melanoma surgery (Wide local excision and SLNB). The identities of patients were protected by anonymizing data to remove all HIPAA identifiers. Patient information and relevant data was stored on password-protected institution computers behind the institutional firewall. Paper records were stored in locked files inside a locked office.

### Intraoperative Radiotracer Measurements

**FIGURE 1:**
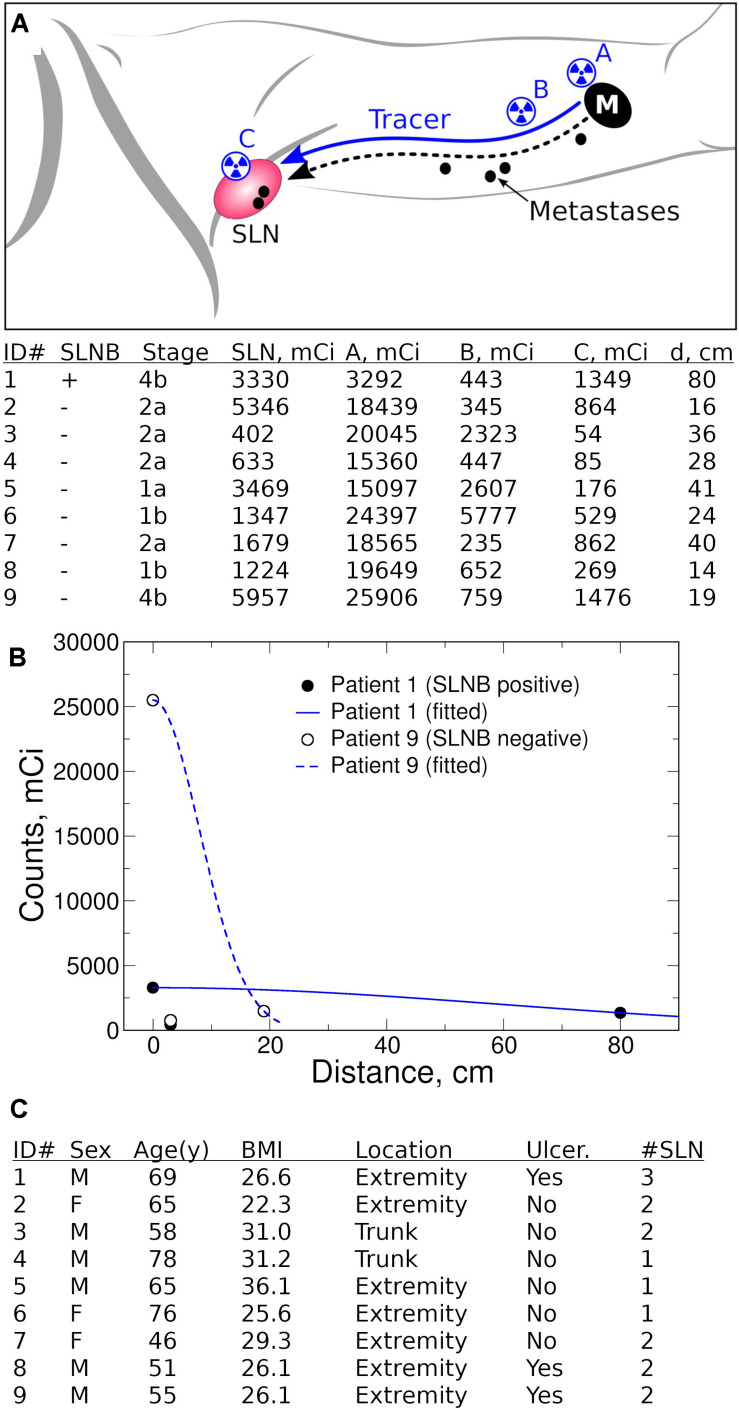
Quantification of lymphatic transport efficiency (LTE). **(A)** Radiotracer (TC-99m SC) LTE is a surrogate for metastatic potential of primary melanoma to tumor-draining lymph nodes (M, primary melanoma, SLN, sentinel lymph node). A single surgeon performed standard intraoperative intradermal peritumoral injection of TC-99m (1–2 mCi) 0.5 ml per quadrant at the edge of the biopsy site/lesion with (A) performed last. The time (Time 0) and gamma count was recorded at location A. After ∼5 min, the surgeon localized the highest gamma count at skin surface of the nodal basin (C), measured the anatomical line on skin connecting (A) and (C), and established the point B 3 cm proximal from (A) along (A–C) line. Stage is T stage (AJCC 8th edition) from preoperative biopsy reviewed at our institution. Gamma counts at points (A), (B), and (C) are measured and recorded along with measurement time. All clinical intraoperative measurements are presented in table, with d, Distance from (A) to (C). **(B)** Fitted gamma count profiles using points (A) and (C). Patient #1 has a wide distribution of counts because of high LTE whereas Patient #9 falls off quickly from the injection site (A) because of poor LTE. **(C)** Clinical characteristics of patient cohort, including demographic and primary tumor data (Ulcer, ulceration status, #SLN, number of SLN examined).

To decrease the effect of variability, a single surgeon performed intraoperative intradermal peritumoral injection of TC-99m (1–2 mCi) 0.5 ml per quadrant at the edge of the biopsy site/lesion with (A) performed last (Figure 1A). Time (Time 0) and gamma count was recorded. The same hand-held gamma probe (Neoprobe, Mammotome) was used to obtain measurements. At 5 min, the surgeon:

1. Localized the highest gamma count at skin surface of the nodal basin (C),
2. Drew a line connecting (A) with (C) on skin and measured and recorded its length,
3. Measured 3 cm proximal from (A) along this line and marked this spot (B),
4. Recorded the time and measured gamma counts at points (B) and (C).

The patient was then prepped and draped (∼5–15 min). The surgeon then repeated measurements of gamma counts at (A), (B), and (C). Although measurement timing is expected to vary within 2–5 min, this variability did not influence the ability to measure Tc-99m SC biodistribution kinetics since they remain steady within the first hour (9). Differences in timing are accounted for in the analysis described below. Standard pathologic analysis was performed on all SLN(s) removed at the time of surgery to determine number and status of SLN(s) excised.

### Quantification of Lymphatic Transport Efficiency

To understand such a complex process as lymphatic metastasis in a rigorous and quantitative manner required an analytical approach using clinical measurements to produce deterministic, reproducible representations of lymphatic transport. Our methodology relies on measurements of tracer permeation, timing and distance (between tumor and SLN) to estimate LTE using a diffusion transport framework. LTE has the same meaning as an apparent diffusion coefficient (ADC), widely used in MRI, that integrates diffusion and convective transport. Although the ADC is a rough approximation of a very complex transport biodistribution and transport problem, this approach offers easy implementation and wide adoption.

By using measured tracer counts at points (A) and (C) 5–10 min following tracer injection, we fitted LTE with the one-dimensional diffusion equation. This equation describes the spread of a constant dose injected at (A) (*x* = 0 at *t* = 0) over time *t* and over distance *x* [the anatomical path (A–C); from the injection site to LN basin]:

$$c\,\left(x,t\right)=\frac{c_{0}}{\sqrt{\left(4\,\pi \,L\,T\,E\,t\right)}}\,\exp\,\left(-\frac{x^{2}}{4\,L\,T\,E\,t}\right)$$

where c(x,t) – measured tracer (gamma counts) at time t (seconds following tracer injection) and distance *x* (cm) from injection site (A), and c_0_ – tracer (counts) at time and site of injection (A).

### Statistical Analysis

Software R and Xmgrace were used for data analysis, correlations, and plotting. Student’s *T*-test was performed comparing clinicopathologic features with estimated transport parameters. Analysis of covariance was used to assess differences between patients regarding these variables. Regression analysis of SLN status and LTE was performed.

## Results

### Lymphatic Transport Efficiency Can Be Determined From Intraoperative Radiotracer Measurements

We examined LTE from the primary tumor to tumor-draining LNs using the radiotracer TC-99m SC as a surrogate for lymphatic transport of melanoma metastases (Figure 1A). Nine patients with clinical Stage I-II melanoma were enrolled as part of an IRB protocol (HMH Pro00021626, PI = Holder). During the SLNB procedure, we performed peritumoral injection of TC-99m SC and measured radiotracer distribution kinetics with a handheld gamma probe. We examined three locations with measured distances between the primary tumor and the tumor-draining nodal basin (Figure 1A). We theorize that the radiotracer concentrates at the injection site A; then after intravasating into lymphatics, the radiotracer is distributed throughout the lymphatic network, causing signal dilution. Lastly, the radiotracer is concentrated again in tumor-draining LNs. The gamma count decrease at point B serves an indirect estimate of dermal lymphatic vessel density. By obtaining measured distances and time, we estimated LTE without reference to the anatomical tumor location, while accounting for distances between the primary tumor and the tumor-draining nodal basin. Intraoperative tracer counts and distances were used to estimate LTE on the basis of an apparent diffusion coefficient. This analysis was achieved by fitting LTE into diffusion equations (Figure 1B).

### Lymphatic Transport Efficiency May Predict Sentinel Lymph Node Status in Melanoma

**FIGURE 2:**
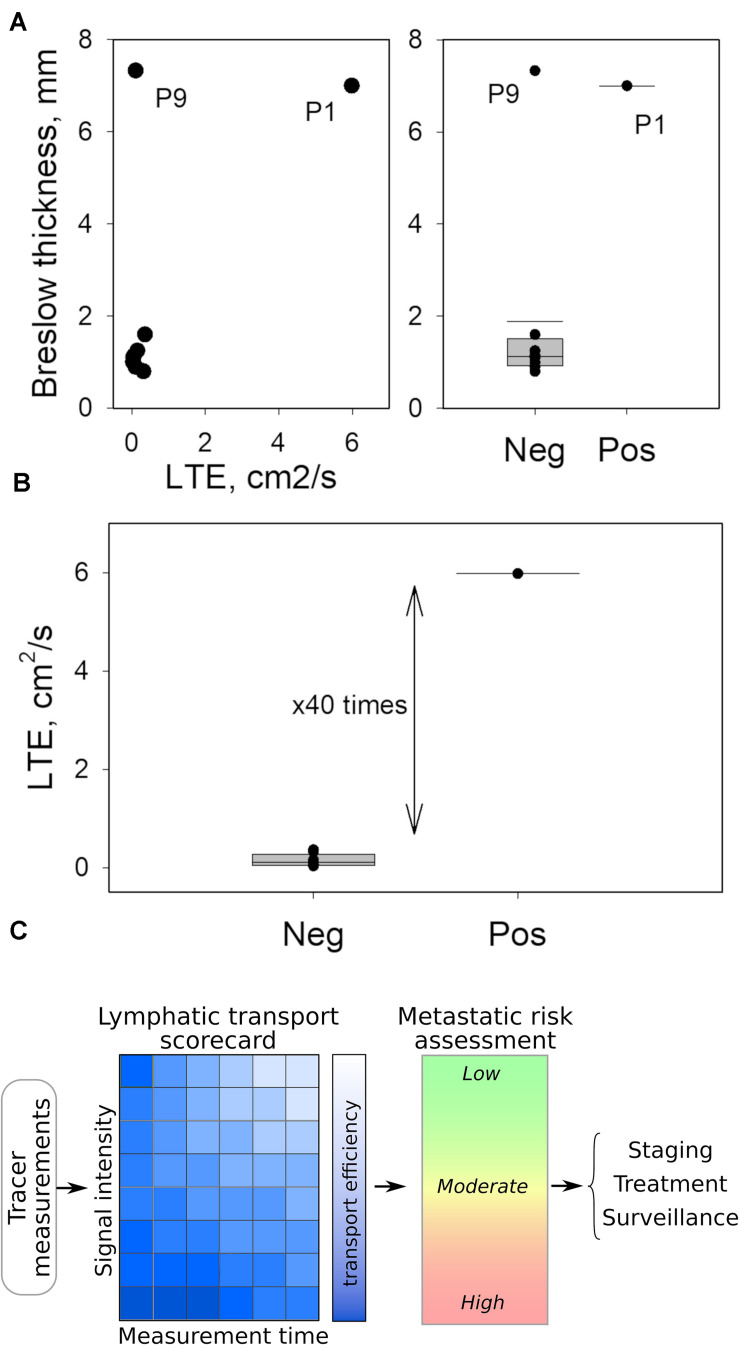
Quantitated LTE as possible biomarker for SLN status. **(A)** Breslow thickness of the primary tumor appears to correlate with LTE except for Patient 9, who had a notably low LTE compared to Patient 1, despite similar Breslow thickness (left panel). Likewise, the Breslow thickness fails to predict the SLN status of Patient 9. **(B)** LTE measurements separate positive and negative SLNs, wherein LTE values differ by almost two orders of magnitude. **(C)** Translating LTE into clinical application can be accomplished by simplified tables or scorecards connecting LTE with time following radiotracer injection and the measured site’s intensity properties. This technique can help clinicians assess metastatic potential practically within minutes in the OR to shape personalized surgical and therapeutic interventions.

We found that LTE correlated with Breslow depth (Figure 2A), with the notable exception of Patient 9 (Stage IIC, T4bN0M0). Importantly, the comparison of Patient 1 (Stage IIID, T4bN3aM0) to Patient 9 (Stage IIC, T4bN0M0) underscores the potential of LTE as a predictive marker of metastatic potential. Both Patient 1 and Patient 9 had thick, ulcerated primary melanomas; however, Patient 9 had much less efficient LTE (Figure 2A) and a negative SLNB (Figure 2A), suggesting LTE is a reliable indicator of SLN status. In fact, the LTE of Patient 1, who had a positive sentinel lymph node biopsy (3/3 SLNs), was 5.98 cm^2^/s compared to 0.14 cm^2^/s – the average LTE of the eight patients with negative SLNs. Hence, lymphatic transport in the node-positive patient is more than 40 times more efficient than in patients with negative SLNB (Figure 2B). At the same time, we found no correlation between LTE and age (*R*^2^ = 0.04, *p* = 0.612), sex (*p* = 0.268), BMI (*R*^2^ = 0.02, *p* = 0.763), dose of TC-99m SC (*R*^2^ = 0.19, *p* = 0.246), *ex vivo* hottest SLN gamma count (*R*^2^ = 0.02, *p* = 0.731) or primary tumor location (extremity v trunk, *p* = 0.268); these findings suggest that LTE is independent of confounding by clinicopathologic variables (Figure 1C). This data indicates that LTE is most related to the likelihood of metastasis. Thus, LTE may serve as a predictive biomarker to determine metastatic risk. This novel technique could be a powerful method to improve patient outcomes.

## Discussion

### False Negative Sentinel Lymph Node Biopsies Necessitate a Better Staging Technique for Cutaneous Melanoma

About 100,000 patients were diagnosed with melanoma and 10,000 died in the United States last year (10). Staging and treatment for melanoma patients without clinical nodal disease is primary tumor excision and SLNB. Besides the histopathologic factors of Breslow thickness and ulceration, the status of the SLN is the most important prognostic indicator to date. However, SLNB is associated with a 15% false negative rate, with the highest false negative rates in patients with thick, ulcerated primary tumors (1, 2). These false negatives result from a number of sources of error, most of which center on human error—identification of the SLN(s) and/or pathologic assessment (3). Furthermore, the SLNB procedure exposes patients to potential morbidity—postoperative seroma, lymphedema, and anesthetic complications. New tools to predict metastatic potential are needed to provide potentially curative treatment.

### Lymphatic Transport Efficiency May Be a Novel Biomarker for Metastasis

Results from our novel analytic approach indicate that LTE is a possible novel biomarker for SLN metastasis. This study suggests the potential to transform the existing standard-of-care, minimally invasive procedure, performed under local anesthesia, into precision medicine that can predict escape of metastatic melanoma from primary tumors to LNs. Compared to the current standard of SLNB, this methodology could provide personalized diagnostics that more accurately and rapidly inform clinical decision-making with decreased cost.

Notably, one of the major limitations of our study is the small sample size. A much larger sample size is required before more definitive conclusions regarding the accuracy and reproducibility of the LTE technique can be proposed.

Multiple factors have been implicated in the modulation of lymphatic transport, including obesity, age, anatomic location, and contraction of surrounding muscles. Because obesity has demonstrated in preclinical models and in patients to cause decreased lymphatic pumping and leakiness of lymphatics (11), we included BMI in our analysis but did not find any significant correlations with LTE. Likewise, increasing age has been associated with a lower incidence of a positive SLNB yet worse disease-specific survival in patients with cutaneous melanoma. Recently, Ecker et al. has proposed that changes in the lymphatic extracellular matrix occur during the aging process that may alter lymphatic permeability allowing metastatic disease to escape from the lymphatic system and directly enter the hematogenous system resulting in visceral or distant metastases (12). Although we did not observe a correlation between age and LTE in our study, we did not evaluate these patients with cross-sectional imaging and have not obtained adequate clinical follow-up to assess for visceral or distant metastases and thus for the association between age, lymphatic permeability, and LTE. Since the lymphatic system is ultimately connected to the systemic circulation through the thoracic duct, the mechanism of metastasis—lymphatic v hematogenous—is likely to be much more complex than the dichotomous approach suggested by our methodology, especially given the inter-related compartments of the lymphatic system and the systemic circulation (13–15). Anatomic location accounts for up to 3–4x difference in the mean lymphatic flow rate from the upper extremity compared to the lower limb (16). One limitation of our analysis is its failure to capture the upper v lower extremity distinction in our data collection. Of note, the primary tumor of patient 1 was located on the plantar surface of the foot while the primary tumor of patient 9 was located on the upper arm. Even accounting for these differences in lymphatic flow rate attributable to anatomic location, the LTE of patient 1 remains 10x faster than that of patient 9. Contraction of surrounding muscles is a factor in lymphatic pumping (14) and thus is a determinant of lymphatic flow. Although we did not have a defined protocol, our patients remained supine for at least 30 min prior to injection of Tc-99m SC from the time of transportation from the preoperative holding area to the operating room and during intubation. One limitation is our inability to account for skeletal muscle contraction, but no patient had a sequential compression device or sphygmomanometer cuff placed on the same extremity as the primary melanoma. In summary, despite multiple potential confounding variables, our novel analysis of LTE remained an independent predictor of SLN in our small patient cohort.

### Predictive Tools That Preserve Tumor-Draining Lymph Nodes May Improve Response to Checkpoint Inhibitors

This finding is the first step for radiotracer LTE to become a prognostic tool to predict metastatic risk and replace sentinel lymph node biopsy in the staging of patients with clinical Stage I-II melanoma. As aforementioned, a study with a larger cohort of patients with melanoma is necessary to investigate the accuracy and reproducibility of LTE as a predictive tool for metastatic risk in cutaneous melanoma. Improving our understanding of the impact of LTE on metastatic potential in melanoma will lead to more accurate and personalized staging (Figure 2C) as well as a new understanding of the importance of transport mechanisms in lymphatic metastasis. In addition, the presence of intact tumor-draining lymph nodes has been associated with improved response to immunotherapy (17). Validation of these findings in a larger cohort of patients can generate a better predictive schema that maintains tumor-draining LNs *in situ*, enabling immunotherapy strategies to improve treatment responses, while decreasing patient morbidity and healthcare costs. Specifically, if our approach can be validated to predict the SLN status with greater than 95% accuracy, the sentinel lymph node could remain in place; only patients with LTE determined to be in an indeterminate range or highly likely to be negative would undergo sentinel lymph node biopsy to verify their staging, given the low likelihood that these patients would require adjuvant immunotherapy. Lastly, this approach exemplifies the importance of multidisciplinary collaboration at the intersection of two seemingly unrelated fields, transport oncophysics and surgical oncology, to facilitate innovation.

## Data Availability Statement

The raw data supporting the conclusions of this article will be made available by the authors, without undue reservation.

## Ethics Statement

The studies involving human participants were reviewed and approved by the Houston Methodist IRB. The patients/participants provided their written informed consent to participate in this study.

## Author Contributions

AH and AZ jointly conceived the study. AH wrote the IRB protocol, obtained intraoperative radiotracer measurements, and collected clinicopathologic data. AZ implemented the analytical model and performed statistical analysis. Both authors analyzed the data, wrote the manuscript, discussed the results and implications, and edited the manuscript at all stages.

## Conflict of Interest

The authors declare that the research was conducted in the absence of any commercial or financial relationships that could be construed as a potential conflict of interest.
